# A large, single‐center, real‐world study of clinicopathological characteristics and treatment in advanced ALK‐positive non‐small‐cell lung cancer

**DOI:** 10.1002/cam4.1059

**Published:** 2017-04-04

**Authors:** Gang Chen, Xi Chen, Yaxiong Zhang, Fang Yan, Wenfeng Fang, Yunpeng Yang, Shaodong Hong, Siyu Miao, Manli Wu, Xiaodan Huang, Youli Luo, Cong Zhou, Run Gong, Yan Huang, Ningning Zhou, Hongyun Zhao, Li Zhang

**Affiliations:** ^1^ Department of Medical OncologySun Yat‐sen University Cancer CenterGuangzhouChina; ^2^ State Key Laboratory of Oncology in South ChinaGuangzhouChina; ^3^ Collaborative Innovation Center for Cancer MedicineGuangzhouChina; ^4^ Department of AnesthesiologySun Yat‐sen University Cancer CenterGuangzhouChina; ^5^ Zhongshan School of MedicineSun Yat‐sen UniversityGuangzhouChina; ^6^ Department of Medical Oncologythe Fifth Affiliated Hospital of Sun Yat‐Sen UniversityZhu HaiChina

**Keywords:** ALK, clinicopathological characteristics, crizotinib, NSCLC, real‐world study

## Abstract

Crizotinib has achieved astonishing success in advanced non‐small‐cell lung cancer (NSCLC) patients harboring anaplastic lymphoma kinase (ALK) rearrangement. However, no real‐world studies described the clinicopathological characteristics and treatment of such patients in China. Patients were consecutively collected from Sun Yat‐sen University Cancer Center. Chi‐square test was applied to explore the relationship between ALK fusion status and metastasis sites. Kaplan–Meier methods and multivariable analyses were used to estimate progression‐free survival (PFS). A total of 291 advanced NSCLC patients (ALK (+), *N* = 97; both ALK & epidermal growth factor receptor (EGFR) (‐), *N* = 194) were enrolled. The occurrence of brain metastasis in ALK‐positive patients was significantly higher than double‐negative ones both at baseline (26.5% vs. 16.5%, *P* = 0.038) and during treatment (25.8% vs. 11.9%, *P* = 0.003), but opposite for pleural effusion (6.2% vs. 26.9%, *P* < 0.001 at baseline; 3.1% vs. 10.3%, *P* = 0.031 during treatment). ALK‐positive patients of 53.6% used crizotinib, whereas others only received chemotherapy (37.1%) or supportive care (9.3%). Usage of crizotinib prolonged PFS compared with chemotherapy in ALK‐positive patients (median PFS 17.6 m vs. 4.8 m, *P* < 0.001). ALK‐positive NSCLC had more brain metastasis and less pleural effusion than double‐negative ones. Crizotinib showed better PFS than chemotherapy in advanced ALK‐positive NSCLC at any line. However, half advanced ALK‐positive patients never received crizotinib, which was grim and need improving.

## Introduction

Non‐small‐cell lung cancer (NSCLC) is the leading cause of cancer mortality 1. Although chemotherapy plays a central role in the treatment of non‐small‐cell lung cancer, small molecular tyrosine kinase inhibitors (TKIs) targeting specific driven mutations have resulted in favorable overall response rate (ORR), progression‐free survival (PFS), and the improvement of quality of life 2, 3, 4, 5, 6, 7.

Fusion of the Echinoderm microtubule‐associated protein like‐4 (EML4) and anaplastic lymphoma kinase (ALK) is one of the most representative druggable targets in NSCLC. Other druggable targets include fusion of the Kinesin family member 5B (KIF5B) and Ret protooncogene (RET), fusion of the Sequestosome 1 (SQSTM1) and ALK, and so on. The fusion protein is highly oncogenic both in vitro and vivo, resulting in constitutive ALK pathway activation and ultimately cancer development 8, 9. Several clinical trials have demonstrated the remarkable efficacy of crizotinib, which made the US Food and Drug Administration (FDA) and European Medicines Agency (EMA) accelerate the approval of it 8, 10, 11. Up to now, crizotinib has become the first‐line option in ALK‐positive advanced NSCLC patients due to the significant prolonged PFS compared with first‐line standard chemotherapy 6.

Currently, some studies have shown that patients harboring the EML4‐ALK rearrangement have distinct clinical characteristics 12, 13, 14, 15. For example, a study showed that patients most likely to harbor EML4‐ALK were young, never/light smokers with adenocarcinoma 14. However, there were few real‐world studies focused on clinicopathological characteristics and treatment in advanced ALK‐positive NSCLC patients. Some oncologists recognized that ALK‐positive patients were easier to have brain metastasis in clinical. However, we have no evidence‐based study comparing the metastatic site between the ALK‐positive advanced NSCLC patients and the both ALK‐negative and epidermal growth factor receptor (EGFR)‐negative ones. Furthermore, the treatment of crizotinib in ALK‐positive patients in the real world is still unclear in China.

Therefore, we carried out this large, single‐center, real‐world study to investigate the specific clinicopathological characteristics of ALK‐positive advanced NSCLC compared with ALK‐negative ones, and to give more clinical evidence of the treatment of crizotinib in ALK‐positive patients in the real world.

## Methods

### Patient and sample collection

This real‐world study was aim to find out the specific clinicopathological characteristics of ALK‐positive NSCLC patients compared with those patients who were both ALK‐negative and EGFR‐negative. Based on the research objective, we retrospectively collected consecutive ALK‐positive patients from 6th Jan 2010 to 26th Apr 2016. In addition, we choose the consecutive double‐negative patients from 10th Jan 2012 to 25th Apr 2014 as comparison group. All the patients who met the following criteria were retrospectively enrolled: (1) histologically or cytologically proven NSCLC patients by Department of Pathology of Sun Yat‐sen University Cancer Center(SYSUCC); (2) aged 18 years old or older; (3) able to provide informed consent; and (4) available and sufficient tumor tissues (biopsy or surgical specimen) for genomic analysis (these specimens were obtained from two sources: fresh‐frozen tumor samples from the Biobank of SYSUCC and formalin‐fixed, paraffin‐embedded (FFPE) tissue submitted to the Department of Pathology). The objective tumor response was determined by independent professional physicians according to RECIST v1.1 (Response Evaluation Criteria in Solid Tumors, version 1.1). PFS was defined as the time from beginning of crizotinib treatment to the first radiographic proof of progressive disease (PD) or death from any causes. The study was approved by ethical committee of SYSUCC. Informed consent was obtained from each participant before the acquisition of tumor tissue and the treatment.

### Genomic analysis

EML4‐ALK rearrangements were detected by means of Fluorescence in situ Hybridization (FISH) using a break‐apart probe to the ALK gene (Vysis LSI ALK Dual Color, Break Apart Rearrangement Probe; Abbott Molecular) as per manufacturer's instructions. At least 100 representative tumor cells were counted. The results obtained by FISH were analyzed using an Olympus fluorescence microscope equipped with orange, green, and 49,6‐diamidino‐2‐phenylindole filters. Images were captured using the Video Test Image Analysis System. FISH‐positive cases were defined as 15% of the tumor cells that showed a split red and green signal and/or an isolated (single) red signal. Otherwise, the specimen was classified as ALK FISH negative. EGFR mutations were detected using PCR‐based direct sequencing of exons 18–21 described as following. Briefly, genomic DNA was extracted from either tumors embedded in paraffin blocks or from fresh‐frozen tumors. PCR amplification was done using HotStarTaq DNA polymerase (Qiagen Inc., Valencia, CA) with a forward primer (59‐GGATCGGCCTCTTCATGC‐39) and a reverse primer (59‐TAAAATTGATTCCAATGCCATCC‐39). PCR products were sequenced directly using Applied Biosystems PRISM dye terminator cycle sequencing method (Perkin‐Elmer Corp., Foster City, CA) with ABI PRISM 3100 Genetic Analyzer (Applied Biosystems, Foster City, CA). Any in‐frame deletions in exon 19 or point mutations in exon 21 (L858R or L861Q substitutions), which confer sensitivity to EGFR‐TKIs therapy, were considered as EGFR mutant. Clinical characteristics including age at diagnosis, gender, smoking history, pathological types, and cancer stage. In addition, the first‐ or second‐line therapeutic regimen and the progression survival time after first‐line treatment were needed to do survival analysis.

### Statistical analysis

All the statistical analysis was performed using SPSS 22.0 for Windows (IBM, Armonk, NY). Chi‐square test (or Fisher exact test) and independent‐samples *t*‐test were applied to explore the association between the clinicopathological variables and EML4‐ALK fusion status and the relationship between ALK fusion status and metastasis sites, for categorical and continuous data, respectively. Kaplan–Meier methods were used to estimate the PFS survival curves, and multivariable analyses were performed to assess survival difference. The prognostic results were reported with hazard ratio (HR) and its 95% confidence interval (CI). HR < 1 implied a lower risk of progression or death for patients. A two‐sided *P* < 0.05 was considered statistically significant.

## Results

### Patients’ characteristics

From 6th Jan 2010 to 26th Apr 2016, 2650 consecutive patients who were pathologically diagnosed with non‐small‐cell lung cancer in SYSUCC were screened. After selecting by inclusive criteria, 97 advanced ALK‐positive NSCLC patients were included. For the control group, we screened 1377 consecutive NSCLC patients in SYSUCC from 10th Jan 2012 to 25th Apr 2014, 194 advanced both ALK‐ and EGFR‐negative patients were enrolled. The clinical characteristics were summarized in Table**** 1. The median age at diagnosis was 50 years (range: 19–83 years). Among them, 40.5% were females, 59.8% were never smokers, 85.2% had adenocarcinoma, and 81.1% were stage IV.

**Table 1:** Clinicopathological characteristics of patients with advanced NSCLC and their relationship with EML4‐ALK rearrangement status

Characteristics	Total *N* (%)	EML4‐ALK rearrangement		*P* ‐value
		ALK(+)	ALK(−) & EGFR(−)	
Patients (*n*)	291	97	194	
Age at diagnosis (years)				0.003
<60 year	222 (76.3)	84 (86.6)	138 (71.1)	
≥60 year	69 (23.7)	13 (13.4)	56 (28.9)	
Gender (*n*)				0.151
Female	118 (40.5)	45 (45.9)	73 (37.6)	
Male	173 (59.5)	52 (53.1)	121 (62.4)	
Smoking status				0.005
No	174 (59.8)	69 (70.4)	105 (54.1)	
Yes	117 (40.2)	28 (28.6)	89 (45.9)	
Drinking status				0.317
No	227 (78.0)	79 (81.4)	148 (76.3)	
Yes	64 (22.0)	18 (18.6)	46 (23.7)	
Pathology status				0.026
ADC	248 (85.2)	89 (90.8)	159 (82.0)	
Non‐ADC	43 (14.8)	8 (8.2)	35 (18.0)	
Stage				<0.001
IV	236 (81.1)	64 (65.3)	172 (88.7)	
Recurrence	55 (18.9)	33 (34.7)	22 (11.3)	

Nonadenocarcinoma means the other types of NSCLC except for adenocarcinoma, which includes squamous cell carcinoma, adenosquamous carcinoma, large‐cell carcinoma, and lymphoepithelioma‐like carcinoma.

ADC, adenocarcinoma; non‐ADC, nonadenocarcinoma; ALK(‐)&EGFR(‐), patients who do not harbor either EML4‐ALK rearrangement or EGFR mutation; NSCLC, non‐small‐cell lung cancer.

### Comparison of clinicopathological data between ALK‐positive patients and double‐negative ones

Patients with EML4‐ALK rearrangements were significantly younger at diagnosis than EGFR and ALK double‐negative patients (median age, 45 vs. 54.5 years; *P* < 0.001). Never smokers were more likely to harbor EML4‐ALK rearrangements than smokers (67.3% vs. 51.0%; *P* = 0.005). Female did not tend to have more chance to harbor EML4‐ALK than male (45.9% vs. 37.6%; *P* = 0.151). A higher percentage of adenocarcinoma was found in patients harboring EML4‐ALK compared with double‐negative ones (90.8% vs. 80.2%; *P* = 0.026). Patients with recurrence had a larger proportion in EML4‐ALK‐positive group compared with double‐negative group (33.7% vs. 11.3%; *P* < 0.001). More detailed results were shown in Table 1.

### Comparison of the metastatic sites between ALK‐positive patients and double‐negative ones

The results of metastatic site comparison between two groups were shown in Table 2. EML4‐ALK‐positive patients tended to have more probability of brain metastases than the double‐negative ones, both at baseline (26.5% vs. 16.5%; *P* = 0.038) and in treatment (25.8% vs. 11.9%; *P* = 0.003). For the analysis stratified by the number of brain metastasis, single brain metastases occurred more frequently in ALK‐positive patients at baseline than both ALK‐ and EGFR‐negative group (11.3% vs. 4.6%; *P* = 0.033), while multiple brain metastasis tended to occur in ALK‐positive patients more often than double‐negative patients during treatment (24.7% vs. 8.2%; *P* < 0.001). Unlike brain metastases, we found no significant difference between ALK‐positive and double‐negative patients of bone metastases either at baseline or in the course of treatment. One thing to be noticed is that patients in ALK‐positive group had more multiple bone metastatic sites than double‐negative group through stratified analysis (17.5% vs. 9.8%; *P* = 0.026). Besides, patients with ALK rearrangement had less chance to get hepatic metastasis than double‐negative patients at baseline (5.2% vs. 13.4%; *P* = 0.032). However, in treatment, there was no significant difference between the two groups (16.5% vs. 10.8%; *P* = 0.171). For adrenal metastasis, no significant difference was found between two groups, no matter at baseline (11.2% vs. 11.3%; *P* = 1.000) or in treatment (2.1% vs. 6.7%; *P* = 0.092). As for malignant pleural effusion, ALK‐positive patients were significantly lower than double‐negative ones, both at baseline (6.2% vs. 26.9%; *P* < 0.001) and in treatment (3.1% vs. 10.3%; *P* = 0.031). It is well known that nearly all EML4‐ALK rearrangement occurs in lung adenocarcinoma 15. In order to adjust for confounding effect of pathological types, we made extra analyses of comparison of the metastatic sites between ALK‐positive patients and double‐negative ones only in adenocarcinoma patients. Similar results were shown in Table S1.

**Table 2:** The comparison of metastatic sites between ALK‐positive patients and double‐negative ones

Metastatic site	ALK(+)		ALK(‐)&EGFR(‐)		*P* ^&^/*P* ^#^
	Baseline *N* (%)	In treatment *N* (%)	Baseline *N* (%)	In treatment *N* (%)	
Brain M	26 (26.5)	25 (25.8)	32 (16.5)	23 (11.9)	0.038/0.003
Single	11 (11.3)	1 (1.0)	9 (4.6)	7 (3.6)	0.033/0.205
Multiple	15 (15.5)	24 (24.7)	23 (11.9)	16 (8.2)	0.389/<0.001
Bone M	25 (25.5)	20 (20.6)	58 (29.9)	29 (14.9)	0.463/0.223
Single	15 (15.5)	3 (3.1)	27 (13.9)	10 (5.2)	0.723/0.422
Multiple	10 (10.3)	17 (17.5)	31 (16.0)	19 (9.8)	0.059/0.026
Hepatic M	5 (5.2)	16 (16.5)	26 (13.4)	21 (10.8)	0.032/0.171
Adrenal M	11 (11.2)	2 (2.1)	22 (11.3)	13 (6.7)	1.000/0.092
Pleural effusion	6 (6.2)	3 (3.1)	52 (26.9)	20 (10.3)	<0.001/0.031

ALK(−)&EGFR(−), patients who do not harbor either EML4‐ALK rearrangement or EGFR mutation; Brain M, brain metastasis; Bone M, bone metastasis; Hepatic M, hepatic metastasis; Adrenal M, adrenal metastasis; *P* ^&^, *P*‐value of comparison at baseline; *P* ^#^, *P*‐value of comparison in treatment.

Baseline means that the metastasis existed at the time of diagnosis; in treatment refers to the new metastasis occurred in the course of treatment or previous metastasis progressed.

### PFS analyses and subgroup analyses

**Figure 1:**
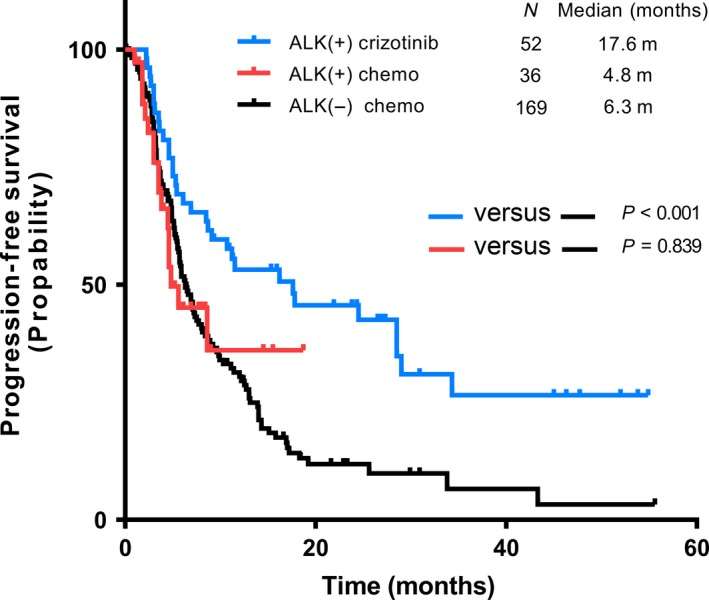
Comparison of PFS among crizotinib/chemotherapy in ALK‐positive patients and chemotherapy in ALK‐negative patients. PFS, progression‐free survival.

**Figure 2:**
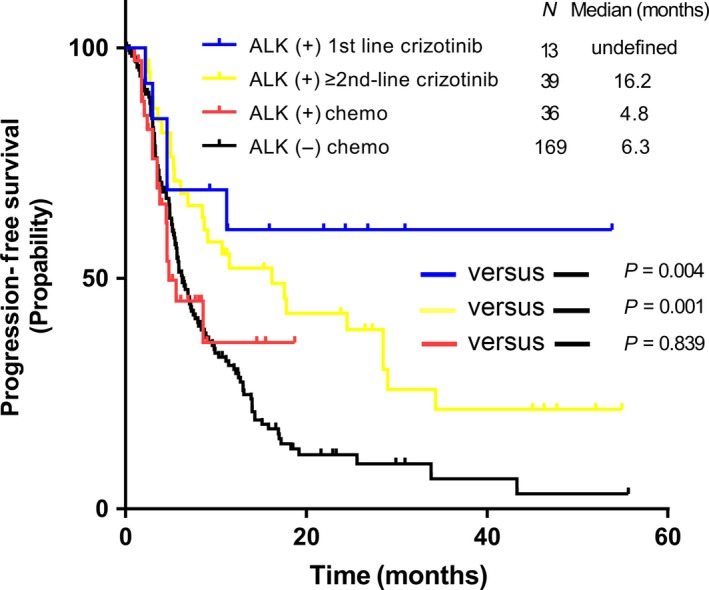
Comparison of PFS among first‐line crizotinib/≥second‐line crizotinib/chemotherapy in ALK‐positive patients and chemotherapy in ALK‐negative patients. PFS, progression‐free survival; ALK, anaplastic lymphoma kinase.

**Figure 3:**
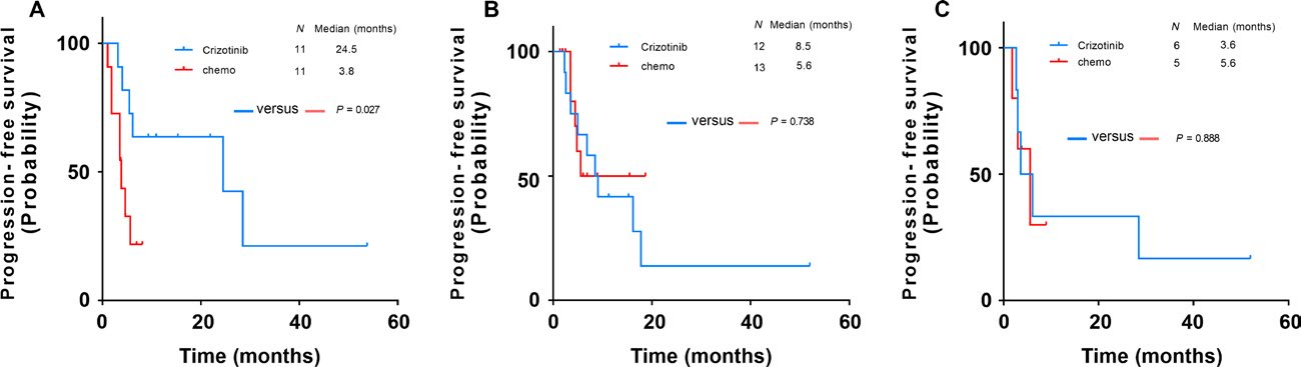
Comparison of PFS between crizotinib and chemotherapy stratified by different metastasis subgroup in ALK‐positive patients. A, in brain metastasis subgroup; B, in bone metastasis subgroup; C, in adrenal metastasis subgroup; PFS, progression‐free survival; ALK, anaplastic lymphoma kinase.

We noticed that only 52 patients (53.6%) used crizotinib in our consecutive enrolled ALK‐positive patients, while others only received chemotherapy (36 patients, 37.1%) or supportive care (9 patients, 9.3%). It revealed that the PFS had significant differences among ALK‐positive patients treated with crizotinib (median PFS 17.6 m), ALK‐positive patients using chemotherapy (median PFS 4.8 m), and ALK‐negative (EGFR&ALK double‐negative) patients using chemotherapy (median PFS 6.3 m) (Fig. 1). The median follow‐up time for the above three groups was 27.3 m, 6.9 m, and 18.5 m, respectively. After dividing ALK‐positive patients taking crizotinib into first‐line crizotinib group and ≥ second‐line crizotinib group, we found numerical PFS benefit in first‐line crizotinib group compared to ≥ second‐line crizotinib group. However, both first‐line crizotinib group and second‐line crizotinib group had significant PFS benefit when they were compared with double‐negative patients using chemotherapy (Fig. 2 **)**. The results of univariate and multivariate analysis of PFS using the Cox regression model were shown in Table 3. After taking other clinical characteristics (Age, Gender, Smoking status) into consideration, crizotinib treatment was the independent factor for better PFS (HR: 0.487; 95% CI: 0.255–0.932; *P* = 0.030). Subgroup analyses showed the comparison of PFS between usage of crizotinib and chemotherapy in ALK‐positive patients stratified by different metastatic site at baseline. It showed that patients taking crizotinib had significant longer PFS than those using first‐line chemotherapy in brain metastasis subgroup (Fig. 3A). In subgroups of bone and adrenal metastasis, the PFS in patients taking crizotinib tended to be longer than those receiving first‐line chemotherapy, although statistical significance was not achieved (Fig. 3B and C). The subgroup results of univariate and multivariate analysis were shown in Tables S2–S4.

**Table 3:** Univariate and multivariate analysis of PFS in ALK‐positive NSCLC patients

Parameter		*N*	Univariate			Multivariate		
			HR	95% CI	*P*‐value	HR	95% CI	*P*‐value
Age			0.427	0.132–1.375	0.154	0.451	0.138–1.478	0.189
<60 year	RC	78						
≥60 year		10						
Gender			0.710	0.402–1.254	0.238	0.763	0.413–1.408	0.387
Female	RC	39						
Male		49						
Smoking			1.300	0.689–2.453	0.418	1.196	0.609–2.351	0.603
No	RC	61						
Yes		27						
Treatment			0.535	0.284–1.009	0.053	0.487	0.255–0.932	0.030
Chemo	RC	36						
Crizotinib		52						

Chemo, chemotherapy; HR, hazard ratio; 95% CI, 95% confidence interval; NSCLC, non‐small‐cell lung cancer; PFS, progression‐free survival; RC, the reference category.

## Discussion

NSCLC was partially characterized by driver mutation‐defined molecular subsets, each with distinct clinicopathological features and potentials for targeted therapies 16. In our study, ALK‐positive patients were younger at diagnosis, and more of them were never smokers and with adenocarcinoma compared with both ALK‐ and EGFR‐negative patients. It was notable that in metastatic site comparison, ALK‐positive patients tended to have more probability of occurring brain metastasis in the course of disease compared with double‐negative patients, which was still statistically significant after picking lung adenocarcinoma patients for separate analysis. In addition, we also found that ALK‐positive patients tended to have lower probability of occurring malignant pleural effusion than negative ones. Besides, in survival analysis, usage of crizotinib prolonged PFS compared with usage of chemotherapy in ALK‐positive patients. Moreover, first‐line crizotinib showed numerical PFS benefit compared with ≥ second‐line crizotinib.

Our study showed that brain metastasis tended to occur more frequently in ALK‐positive advanced NSCLC patients in the course of treatment, which was similar to the previous study 17. In that study, 45% of patients who harbored ALK rearrangement with follow up had progressive brain metastasis at the time of death. It might be explained by the following reason. Patients with ALK fusion were likely to use crizotinib that extended survival due to the better systemic control of disease, which to some extent contributed to the higher opportunities of occurring brain metastasis in ALK‐positive patients. Moreover, we noticed a significant survival benefit in ALK‐positive patients with brain metastasis who had took crizotinib, which was consistent with the results in the pool analysis of PROFILE‐1005 and PROFILE‐1007, and kept up with the outcome of PROFILE‐1014 18, 19. The advantage of crizotinib over chemotherapy indicated that among the ALK‐positive NSCLC patients with brain metastasis, crizotinib should be preferred. However, we could not neglect the result that ALK‐positive NSCLC patients had more occurrence of brain metastasis than double‐negative ones in the treatment of disease, which largely attribute to the poor penetration of crizotinib 20, 21.

Based on the current knowledge, next‐generation TKIs have demonstrated increased central nervous system (CNS) penetration and activity in early clinical trials. A phase I/II trial of alectinib in crizotinib‐refractory ALK‐positive NSCLC included 16 patients with baseline measurable CNS disease, at the time of analysis, showed 4 (25%) of 16 patients had achieved a complete CNS response and 8 (50%) had achieved a partial response, Median duration of CNS response was 11.1 months (95% CI: 5.8–11.1). Sixteen (100%) patients achieved disease control in the CNS 22. Similarly, a separate phase II trial of alectinib for crizotinib‐refractory disease reported a complete response in 43% of patients with baseline brain metastases and no prior radiation 23. The intracranial response in this trial was 57%, and at 12 months, the cumulative rate of CNS progression (25%) was lower than non‐CNS progression (33%), suggesting a potential change in the patterns of failure observed with crizotinib. Other next‐generation ALK‐targeted TKIs have also demonstrated encouraging results. A phase I study of ceritininb showed intracranial response rates of 36% and 63% in measurable baseline brain metastases with and without exposure to prior ALK inhibitor therapy, respectively 24. Brigatinib also demonstrated outstanding control of CNS metastasis in a phase I/II trial 25. In this study, the ORRs of patients with measurable (≥10 mm) brain lesions were 53%, and the intracranial disease control rate was 87%. For all patients with an intracranial response, median duration of intracranial response was 18.9 months. The increased CNS penetration and activity of next‐generation ALK‐TKIs indicate that the treatment of patients with brain metastasis could gradually switch from the whole‐brain radiation therapy (WBRT) plus first‐generation TKIs to stereotactic radiosurgery (SRS) combined with next‐generation TKIs, which will improve health‐related quality of life and decrease the risk of decline in learning and memory function 26, 27.

About 30–40% of patients affected by NSCLC develop, during the course of their disease, bone metastases 28. A large number of patients with bone metastasis will subsequently experience skeletal‐related events (SREs) 29, which refer to a collection of adverse events associated with bone metastasis, including pathologic fractures, the requirement for surgery or radiotherapy, spinal cord and nerve root compression, and hypercalcemia of malignancy 30. What we found was that both ALK‐positive patients and ALK‐negative patients had similar occurrence of bone metastasis in the course of disease. Interestingly, for patients in ALK‐positive group more multiple bone metastatic sites occurred than double‐negative group, which suggested that advanced ALK‐positive NSCLC patients might have a higher possibility of experiencing SREs in the course of disease, compared with the ALK‐negative patients. This finding also probably attributes to the extended survival benefit of using crizotinib in ALK‐positive patients. The results remind clinicians that a whole‐process management of bone metastasis should be applied into ALK‐positive patients.

One previous study found that ALK‐positive patients were predisposed to liver metastasis compared to the patients who were ALK negative and did not harbor the EGFR and KRAS mutation 31, which was not validated in our study. Here, are the possible reasons for the difference. Firstly, the sample size of patients with EML4‐ALK rearrangement varied among different studies and all data were retrospectively collected, resulting in potential bias. Secondly, although all the patients enrolled were ALK positive among different studies, the discrepancy in demographic characteristics and treatment might have influence on the outcome. In the future, multicenter study might figure out whether ALK‐positive patients had a higher incidence of liver metastasis.

Interestingly, our result also presented that ALK‐positive patients had significantly lower incidence of pleural effusion than both ALK‐ and EGFR‐negative patients. Based on the results that ALK‐positive patients were inclined to have brain and bone metastasis, we hypothesized that ALK‐positive patients tended to form distant metastasis through hematogenous spread, while both ALK‐ and EGFR‐negative patients tended to invade local region by lymphatic vessels. Further validation researches in basic science are warranted to disclose the truth beneath the phenomenon.

As for survival analysis, similar to the previous clinical trials 6, 11, 32, our real‐world study showed that ALK‐positive patients taking crizotinib had a significantly longer PFS than the ALK‐positive patients and both ALK‐ and EGFR‐negative patients who received chemotherapy. One thing to be noticed was that the PFS results in our study was longer than the newest result from PROFILE‐1029, a phase III randomized controlled trial which compared crizotinib with chemotherapy in the first‐line treatment of east Asian ALK‐positive NSCLC patients 33. In this study, PFS of ALK‐positive patients taking crizotinib was 11.1 months. There are several reasons for the discrimination. Firstly, according to one previous study 34, different EML4‐ALK variants might have impact on the efficacy of crizotinib, of which EML4‐ALK variant 1 (v1) had better efficacy of crizotinib than other variants. One recent retrospective study also got the same conclusion, in which EML4‐ALK v1 patients had significantly longer PFS than non‐EML4‐ALK variants (median 31.1  vs. 5.7 months, *P* = 0.003) 35. Hence, we presumed that the higher percentage of EML4‐ALK v1 probably account for the prolonged PFS. Secondly, due to the nature of retrospective study, the result might be influenced by some confounding factors.

From the analytic results, we found out that there were still a great number of ALK‐positive patients who did not take crizotinib, probably because of the high expense of target therapy. This finding revealed that, the treatment option might be influenced by many nonmedical factors in the real world, which to a certain extent influenced the therapeutic efficacy of ALK‐positive patients. Besides, better PFS benefit in ALK‐positive patients who took first‐line crizotinib gave a hint that the use of crizotinib should be initiated as first‐line treatment.

For subgroup survival analysis divided by metastatic site at baseline, ALK‐positive NSCLC patients with brain metastasis could benefit from crizotinib compared with chemotherapy. This result was consistent with the recent result from clinical trial PROFILE‐1014 19, and multivariate analysis showed positive results, which suggested that the usage of crizotinib in the management of ALK‐positive NSCLC brain metastasis. We failed to find significant difference in other subgroup due to the limitation of sample size. Therefore, for the further investigation of the efficacy of crizotinib for the other metastatic sites, an enhancement of the sample size enrolled in each subgroup was the key element to reduce bias.

Our study had several limitations. Firstly, this is a single‐institution study. However, we enrolled consecutive NSCLC patients in our hospital. We believe that the consecutive patients could properly represent the treatment situation in our hospital. Secondly, due to the limited sample size, the survival analysis in some subgroups failed to show significant difference. But through the tendency of survival curves, we could hypothesize that increasing the sample size or conducting prospective cohort study could further elucidate the therapeutic value of crizotinib in ALK‐positive patients compared with chemotherapy.

In summary, our real‐world study showed that patients harboring EML4‐ALK rearrangement tended to be younger at diagnosis, and more of them were nonsmokers and pathological diagnosed with adenocarcinoma compared with those without ALK rearrangement and EGFR mutation. Besides, ALK‐positive NSCLC had more brain metastasis and less pleural effusion than double‐negative ones. Moreover, crizotinib showed better PFS than chemotherapy in advanced ALK‐positive NSCLC, which should be recommended, especially as first‐line choice. However, almost half of advanced ALK‐positive patients did not take crizotinib, which reflected a serious situation in the treatment of ALK‐positive NSCLC in China.

## Conflict of Interest

The authors have declared no conflicts of interest.

## Supporting information


**Table S1.** Association of metastatic site of patients with lung adenocarcinoma at baseline and in treatment with EML4‐ALK rearrangement.

**Table S2.** Univariate and multivariate analysis of PFS in ALK‐positive NSCLC patients with brain metastasis.

**Table S3.** Univariate and multivariate analysis of PFS in ALK‐positive NSCLC patients with bone metastasis.

**Table S4.** Univariate and multivariate analysis of PFS in ALK‐positive NSCLC patients with adrenal metastasis.
