# Supplementary material for: A large, single‐center, real‐world study of clinicopathological characteristics and treatment in advanced ALK‐positive non‐small‐cell lung cancer
Source: Cancer Med. 2017 Apr 4;6(5):953–61. doi: 10.1002/cam4.1059 (PMC5430086; doi:10.1002/cam4.1059)
Supplement: Supplementary file 1 — Table S1. Association of metastatic site of patients with lung adenocarcinoma at baseline and in treatment with EML4‐ALK rearrangement. [file CAM4-6-953-s001.docx]

**Supplemental Table 1. Association of metastatic site of patients with lung adenocarcinoma at baseline and in treatment with EML4-ALK rearrangement.**

| **Metastatic site** | **ALK(+)** | |  | **ALK(-)&EGFR(-)** | | **P^&^/P^#^** |
| --- | --- | --- | --- | --- | --- | --- |
|  | **Baseline N(%)** | **In treatment N(%)** |  | **Baseline N(%)** | **In treatment N(%)** |  |
| **Brain M** | 25(28.1) | 24(26.9) |  | 30(18.9) | 18(11.3) | 0.094/0.002 |
| **Single** | 11(12.4) | 1(1.1) |  | 8(5.0) | 4(2.5) | 0.037/0.454 |
| **Multiple** | 14(15.7) | 23(25.8) |  | 22(13.8) | 14(8.8) | 0.685/0.004 |
| **Bone M** | 22 (24.7) | 20 (22.5) |  | 46(28.9) | 23(14.5) | 0.476/0.110 |
| **Single** | 13(14.6) | 3 (3.4) |  | 21(13.2) | 10(6.3) | 0.759/0.635 |
| **Multiple** | 9(10.1) | 17 (19.1) |  | 25(15.7) | 13(8.2) | 0.218/0.011 |
| **Hepatic M** | 5(5.6) | 15(16.9) |  | 15(9.4) | 16(10.2) | 0.290/0.121 |
| **Adrenal M** | 10(11.2) | 2(2.2) |  | 17(10.7) | 11(6.9) | 0.895/0.113 |
| **Pleural effusion** | 6(6.7) | 3(3.4) |  | 47(29.6) | 15(9.4) | <0.001/0.078 |

Abbreviations: ALK(-)&EGFR(-), patients who do not harbor either EML4-ALK rearrangement or EGFR mutation; Brain M, brain metastasis; Bone M, bone metastasis; Hepatic M, hepatic metastasis; Adrenal M, adrenal metastasis; P^&^, P-value of comparison at baseline; P^#^, P-value of comparison in treatment.

Note: baseline means that the metastasis existed at the time of diagnosis; in treatment refers to the new metastasis occurred in the course of treatment or previous metastasis progressed.
