# Supplementary material for: A large, single‐center, real‐world study of clinicopathological characteristics and treatment in advanced ALK‐positive non‐small‐cell lung cancer
Source: Cancer Med. 2017 Apr 4;6(5):953–61. doi: 10.1002/cam4.1059 (PMC5430086; doi:10.1002/cam4.1059)
Supplement: Supplementary file 2 — Table S2. Univariate and multivariate analysis of PFS in ALK‐positive NSCLC patients with brain metastasis. [file CAM4-6-953-s002.docx]

**Supplemental Table 2. Univariate and multivariate analysis of PFS in ALK-positive NSCLC patients with brain metastasis.**

| **Parameter** |  |  | **Univariate** | | | **Multivariate** | | |
| --- | --- | --- | --- | --- | --- | --- | --- | --- |
|  |  | **N** | **HR** | **95%CI** | **p-value** | **HR** | **95%CI** | **p-value** |
| **Age** |  |  | 0.034 | 0.000-15.828 | 0.281 | 0 | 0.000- | 0.976 |
| **<60y** | RC | 19 |  |  |  |  |  |  |
| **≥60y** |  | 3 |  |  |  |  |  |  |
| **Gender** |  |  | 1.058 | 0.316-3.544 | 0.927 | 1.299 | 0.354-4.733 | 0.693 |
| **female** | RC | 7 |  |  |  |  |  |  |
| **male** |  | 15 |  |  |  |  |  |  |
| **Smoking** |  |  | 1.211 | 0.332-4.410 | 0.772 | 0.672 | 0.170-2.662 | 0.572 |
| **no** | RC | 17 |  |  |  |  |  |  |
| **yes** |  | 5 |  |  |  |  |  |  |
| **Treatment** |  |  | **0.274** | **0.080-0.935** | **0.039** | **0.118** | **0.027-0.523** | **0.005** |
| **Chemo** | RC | 11 |  |  |  |  |  |  |
| **crizotinib** |  | 11 |  |  |  |  |  |  |

Abbreviations: Chemo, chemotherapy; HR, hazard ratio; 95%CI, 95% confidence interval; NSCLC, non-small-cell lung cancer; PFS, progression-free survival; RC, the reference category.
