# Supplementary material for: A large, single‐center, real‐world study of clinicopathological characteristics and treatment in advanced ALK‐positive non‐small‐cell lung cancer
Source: Cancer Med. 2017 Apr 4;6(5):953–61. doi: 10.1002/cam4.1059 (PMC5430086; doi:10.1002/cam4.1059)
Supplement: Supplementary file 3 — Table S3. Univariate and multivariate analysis of PFS in ALK‐positive NSCLC patients with bone metastasis. [file CAM4-6-953-s003.docx]

| **Parameter** |  |  | **Univariate** | | | **Multivariate** | | |
| --- | --- | --- | --- | --- | --- | --- | --- | --- |
|  |  | **N** | **HR** | **95%CI** | **p-value** | **HR** | **95%CI** | **p-value** |
| **Age** |  |  | 0.389 | 0.050-3.028 | 0.367 | 0.437 | 0.040-4.798 | 0.498 |
| **<60y** | RC | 21 |  |  |  |  |  |  |
| **≥60y** |  | 4 |  |  |  |  |  |  |
| **Gender** |  |  | 1.365 | 0.473-3.942 | 0.565 | 3.572 | 0.907-14.071 | 0.069 |
| **female** | RC | 13 |  |  |  |  |  |  |
| **male** |  | 12 |  |  |  |  |  |  |
| **Smoking** |  |  | 2.407 | 0.652-8.884 | 0.188 | 3.406 | 0.709-16.354 | 0.126 |
| **no** | RC | 17 |  |  |  |  |  |  |
| **yes** |  | 8 |  |  |  |  |  |  |
| **Treatment** |  |  | 1.208 | 0.398-3.663 | 0.739 | 2.054 | 0.555-7.607 | 0.281 |
| **Chemo** | RC | 13 |  |  |  |  |  |  |
| **crizotinib** |  | 12 |  |  |  |  |  |  |

**Supplemental Table 3. Univariate and multivariate analysis of PFS in ALK-positive NSCLC patients with bone metastasis.**

Abbreviations: Chemo, chemotherapy; HR, hazard ratio; 95%CI, 95% confidence interval; NSCLC, non-small-cell lung cancer; PFS, progression-free survival; RC, the reference category.
