# Supplementary material for: A large, single‐center, real‐world study of clinicopathological characteristics and treatment in advanced ALK‐positive non‐small‐cell lung cancer
Source: Cancer Med. 2017 Apr 4;6(5):953–61. doi: 10.1002/cam4.1059 (PMC5430086; doi:10.1002/cam4.1059)
Supplement: Supplementary file 4 — Table S4. Univariate and multivariate analysis of PFS in ALK‐positive NSCLC patients with adrenal metastasis. [file CAM4-6-953-s004.docx]

**Supplemental Table 4. Univariate and multivariate analysis of PFS in ALK-positive NSCLC patients with adrenal metastasis.**

| **Parameter** |  |  | **Univariate** | | | **Multivariate** | | |
| --- | --- | --- | --- | --- | --- | --- | --- | --- |
|  |  | **N** | **HR** | **95%CI** | **p-value** | **HR** | **95%CI** | **p-value** |
| **Age** |  |  | — | — | — | 0.985 | 0.905-1.072 | 0.731 |
| **<60y** | RC | 11 |  |  |  |  |  |  |
| **≥60y** |  | 0 |  |  |  |  |  |  |
| **Gender** |  |  | 0.527 | 0.104-2.660 | 0.438 | 0.680 | 0.087-5.283 | 0.712 |
| **female** | RC | 10 |  |  |  |  |  |  |
| **male** |  | 6 |  |  |  |  |  |  |
| **Smoking** |  |  | 1.461 | 0.278-7.663 | 0.654 | 1.461 | 0.179-11.958 | 0.723 |
| **no** | RC | 11 |  |  |  |  |  |  |
| **yes** |  | 5 |  |  |  |  |  |  |
| **Treatment** |  |  | 0.899 | 0.198-4.073 | 0.890 | 0.933 | 0.196-4.440 | 0.931 |
| **Chemo** | RC | 5 |  |  |  |  |  |  |
| **crizotinib** |  | 6 |  |  |  |  |  |  |

Abbreviations: Chemo, chemotherapy; HR, hazard ratio; 95%CI, 95% confidence interval; NSCLC, non-small-cell lung cancer; PFS, progression-free survival; RC, the reference category.
